# Identification of apoptosis-related key genes and the associated regulation mechanism in thoracic aortic aneurysm

**DOI:** 10.1186/s12872-023-03516-0

**Published:** 2023-09-28

**Authors:** Qi Ma, Long Hu, Yingwan Luo, Miao Wang, Shui Yu, Aidong Lu, Leping Zhang, Huimin Zeng

**Affiliations:** 1 Department of Anesthesiology, Hospital of Chengdu University of Traditional Chinese Medicine, Chengdu, China; 2 XJTLU Wisdom Lake Academy of Pharmacy, Xi’an Jiaotong-Liverpool University, Suzhou, China; 3 Department of Hematology, The First Affiliated Hospital, Zhejiang University School of Medicine, Hangzhou, China; 4 Department of Pediatrics, Peking University People’s Hospital, 11 Xizhimen South Street, Beijing, 100000 China

**Keywords:** Apoptosis, Thoracic aortic aneurysm, Transcriptomics, RNA modification, Bioinformatical analysis

## Abstract

**Background:**

This study investigated the role of apoptosis-related genes in thoracic aortic aneurysms (TAA) and provided more insights into TAA's pathogenesis and molecular mechanisms.

**Material/methods:**

Two gene expression datasets (GSE9106 and GSE26155) were retrieved from the Gene Expression Omnibus (GEO) database. Apoptosis-related genes were obtained from the KEGG apoptosis pathway (hsa04210). Differentially expressed apoptosis-related genes were identified by performing differential expression analysis using limma for TAA blood and tissue samples. GO and KEGG enrichment analysis of the differentially expressed apoptosis genes was performed using the Metascape web tool. The miRNA-mRNA regulatory network was reconstructed using the ENCORI and miRDB databases, and functional enrichment analysis was performed on the related miRNAs using the miEAA tool. The correlation between the expression levels of differentially expressed apoptosis-related genes and genes involved in immune infiltration in TAA was calculated using the CIBERSORT algorithm. The apoptosis modification patterns mediated by differentially expressed apoptosis-related genes were systematically assessed in TAA samples.

**Results:**

A total of 9 differentially-expressed apoptosis-related genes were identified in TAA samples compared with normal samples. 150 miRNAs and 6 mRNAs regulatory networks were reconstructed using the ENCORI and miRDB databases. Immune infiltration analysis revealed that the GZMB had the strongest positive correlation with activated NK cells and the DFFA presented the strongest positive correlation with T cells follicular helper. 3 distinct apoptosis modification patterns mediated by 9 differentially-expressed apoptosis-related genes were identified. They differ in immune characteristics and drug sensitivity, and their biological functions in these subtypes were further studied.

**Conclusions:**

This study identified key apoptosis-related genes related to TAA and evaluated the modification patterns of key apoptosis genes in TAA, providing insights into potential targets and mechanisms of TAA pathogenesis and progression.

## Introduction

Thoracic aortic aneurysms (TAAs) are defined as localized dilations of supradiaphragmatic caused by weakening and dilation of the arterial wall [1]. Many TAAs cases remain undetected until complications such as aortic dissection or rupture occur due to their asymptomatic and indolent characteristics. Due to the diversity of tumor cells, Therapies have not been consistently and substantially improved failing to improve the clinical outcomes of TAA treatment [2]. Despite the progress in the surgical care of TAAs, operative risks still stay high. Patients surviving surgery face a significant risk of postoperative complications such as myocardial infarction, renal failure, stroke, neurological deficit, and paraplegia [3].

Cell death plays a critical role in regulating cell proliferation, maintaining cellular homeostasis, and tumor progression [4]. Apoptosis refers to the autonomous and orderly cell death controlled by genes to maintain homeostasis [5]. Unlike cell necrosis, apoptosis is an active process involving the activation, expression and regulation of a series of genes. Also, it is not a phenomenon of autologous injury under pathological conditions, but a death process actively pursued to better adapt to the living environment. Apoptosis is a process tightly controlled by multiple genes, which are highly conserved between species, such as the Bcl-2 family, caspase family, and oncogenes (e.g., C-myc, tumor suppressor gene P53) [6]. The development of molecular biology techniques has promoted the understanding of the process of apoptosis in a variety of cells, but the exact apoptosis mechanism process remains unclear. Disturbances in the apoptosis process may be directly or indirectly related to the development of many diseases, including tumors, autoimmune diseases, etc. A recent study has revealed the involvement of apoptosis-related pathways and genes in thoracic aortic aneurysms [7]. However, the mechanism of apoptosis in TAAs should be further studied.

Deploying a combination of differential expression and data mining methods, this paper identified differentially expressed apoptosis-related genes and explored their expression mechanism. Moreover, ROC analysis showed that the differentially-express apoptosis-related genes present a relatively high diagnostic value for TAA. In addition, we investigated key miRNAs that might produce a marked effect in TAAs and reconstructed the mRNA-miRNA network to identify key apoptosis-related genes and study the pathogenesis of TAAs at the molecular level. Our findings further suggested a potential association between apoptosis and the immune microenvironment, based on the results of the analysis performed using the CIBERSORT tool, and identified three apoptosis-related gene expression patterns through consensus clustering.

## Materials and methods

### Data mining and pre-processing

GEOquery package of R software was used to download TAAs expression profile dataset GSE9106 from GEO (https://www.ncbi.nlm.nih.gov/geo/) [8]. The GSE9106 dataset included 59 TAAs patients and 34 healthy individuals, who were all included in this study (Table 1). Using mRNA recorded in the HUGO Gene Nomenclature Committee (HGNC) (http://www.genenames.org/) [9], the GSE9106 dataset was annotated to obtain an mRNA expression matrix for TAAs. We obtained 136 apoptosis-related genes for subsequent analysis from KEGG pathway apoptosis (hsa04210) [10]. The GSE26155 dataset included 53 TAAs patients and 13 healthy individuals. Similarly, we obtained the mRNA expression matrix of this dataset for subsequent validation.

**Table 1:** The 9 apoptosis regulators that were found to be differentially expressed between TAAs versus healthy samples

Genes	Name
CASP6	Caspase 6
GZMB	Granzyme B
SPTA1	Spectrin Alpha, Erythrocytic 1
DFFA	DNA Fragmentation Factor Subunit Alpha
BCL2L1	BCL2 Like 1
JUN	Jun Proto-Oncogene, AP-1 Transcription Factor Subunit
PTPN13	Protein Tyrosine Phosphatase Non-Receptor Type 13
GADD45G	Growth Arrest and DNA Damage Inducible Gamma
AKT3	AKT Serine/Threonine Kinase 3

### The landscape of differentially-expressed apoptosis-related genes in TAAs

Differential expression analysis was performed comparing the TAAs group and healthy groups in the GSE9106 dataset. The TAA dataset was screened for DEGs of mRNAs using the limma package [11]. Regarding DEG’s criteria the FDR < 0.05 and | log2FC |> 0.5 were used. Correlation heatmaps were plotted with the corrplot package to visualize the associations of differentially expressed apoptosis-related genes. The GOSemSim package [12] was deployed to perform Gene Ontology semantic similarity analysis of the differentially-expressed apoptosis-related genes and to score the semantic similarity of GO terms in the gene clusters. Gene ontology (GO) and pathway Kyoto Encyclopedia of Genes and Genomes (KEGG) enrichment analyses were performed using the Metascape web tool (https://metascape.org/) [13]. ROC curves were used to assess the diagnostic potential of the differentially-expressed apoptosis-related genes. Then, these findings were validated in the TAA dataset GSE26155. We extracted the expression levels of 9 genes for ROC analysis to assess their diagnostic efficacy. The pROC package was used to generate the ROC curves.

### Reconstruction of mRNA-miRNA interaction network

The ENCORI (https://starbase.sysu.edu.cn/) [14] and the miRDB databases (http://mirdb.org/) [15] were used to predict miRNAs targeting the differentially-expressed apoptosis-related genes. The miRNAs predicted by the ENCORI and the miRDB databases were intersected to obtain mRNA-miRNA interaction networks, which were then visualized using Cytoscape. GO and KEGG enrichment analyses of the differentially-expressed apoptosis-related gene-bound miRNAs were performed using miEAA [16] (https://ccb-compute2.cs.uni-saarland.de/mieaa2/).

### Immune infiltration analysis

The CIBERSORT [17] tool is based on using linear support vector regression to perform deconvolution of the transcriptome expression matrix, thereby estimating the composition and abundance of immune cells in mixed cells [11]. Immune cell data were retrieved from the CIBERSORT official website (https://cibersort.stanford.edu/) and ssGSEA and CIBERSORT tools were deployed to evaluate the correlations between the scores of immune infiltration and the expression levels of the differentially-expressed apoptosis-related genes.

### Unsupervised cluster analysis

Unsupervised cluster analysis was used to identify the regulatory patterns of apoptotic genes in TAA by the ConsensusClusterPlus package utilizing the expression levels of differentially-expressed apoptosis-related genes [18]. In specific, the “pRRophetic” package [19] of R was used to predict the half-maximal inhibitory concentration (IC50) of chemotherapy drugs in different clusters and to infer the sensitivity of the different patients, Comparisons among the various clinicopathological variables were also performed and the differences in tumor immune microenvironment between different clusters of patients were assessed to further explore the associations between the APGs-based TAAs and the clinical features or the local immune status of TAA patients. The gene set enrichment analysis (GSEA) of the gene expression matrix based on different apoptotic gene regulatory patterns was performed by the clusterProfiler package, selecting "c2.cp.kegg.v7.0.symbols.gmt" as a reference gene set, and considering significantly enriched terms and *p*-value was less than 0.1.

### Statistical analysis

All data processing and statistical analysis were conducted using R software, Version 4.1.1. The pROC package [20] was used to perform ROC analysis, and the area under the curve (AUC) was calculated to assess the diagnostic ability of the differentially-expressed apoptosis-related genes. The correlation between the differentially-expressed apoptosis-related genes and the immune infiltration was determined by performing Pearson's correlation analysis. The Wilcoxon rank-sum test was used to compare the differences between groups of continuous data. * *p* < 0.05; ** *p* < 0.01; *** *p* < 0.001; **** *p* < 0.0001.

## Results

### The landscape of gene variation of apoptosis regulators in TAAs

**Fig. 1:**
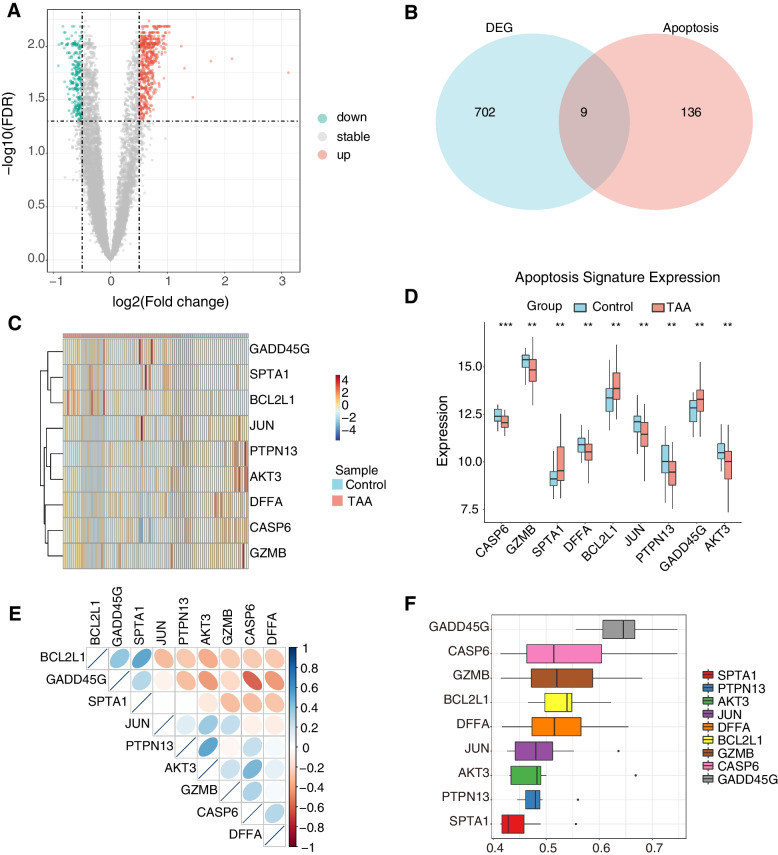
The landscape of gene expression changes of apoptosis regulators in TAAs. **A** TAAs differential expression analysis, where red and blue represented up-regulation and down-regulation, respectively; **B** Venn diagram of DEGs and differentially-expressed apoptosis-related genes; **C** Heatmap of differentially-expressed apoptosis-related gene expression, where red and blue represented TAAs group and healthy group, respectively; **D** Boxplots showing the expression levels of the differentially-expressed apoptosis-related genes, where red and blue represented TAAs group and healthy group, respectively. * *p* < 0.05; ** *p* < 0.01; *** *p* < 0.001; **** *p* < 0.0001; **E** Heatmap showing the correlations among the differentially-expressed apoptosis-related genes, where red and blue represented positive and negative correlations, respectively. Color darkness is relative to the correlation’s strength; **F** Functional similarity analysis using differentially-expressed apoptosis-related genes

**Fig. 2:**
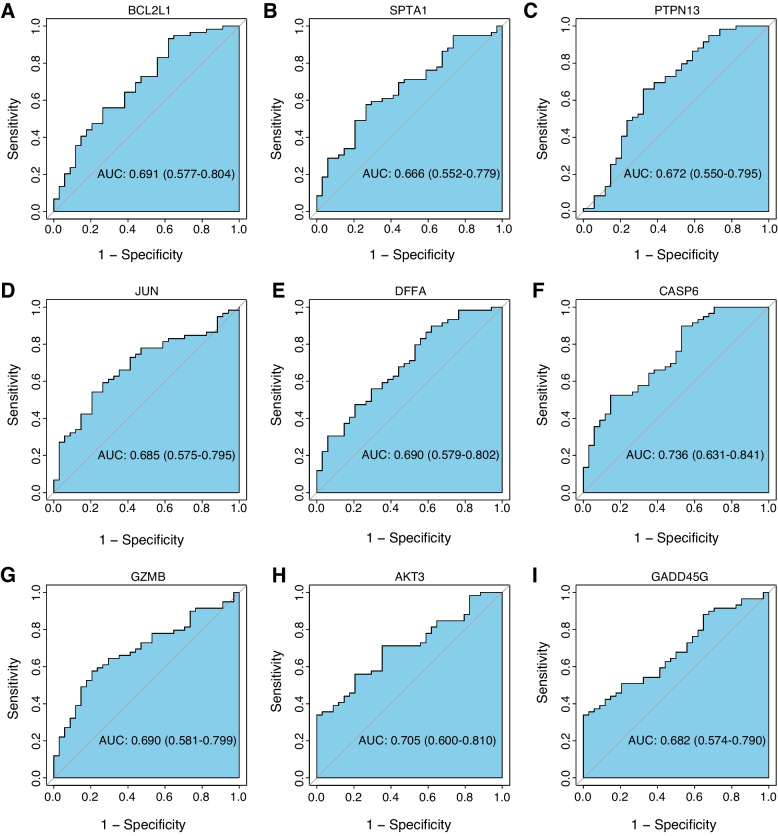
ROC analysis of the differentially-expressed apoptosis-related genes in GSE9106. **A** BCL2L1; **B** SPTA1; **C** PTPN13; **D** JUN; **E** DFFA; **F** CASP6; **G** GZMB; **H** AKT3; **I** GADD45G

**Fig. 3:**
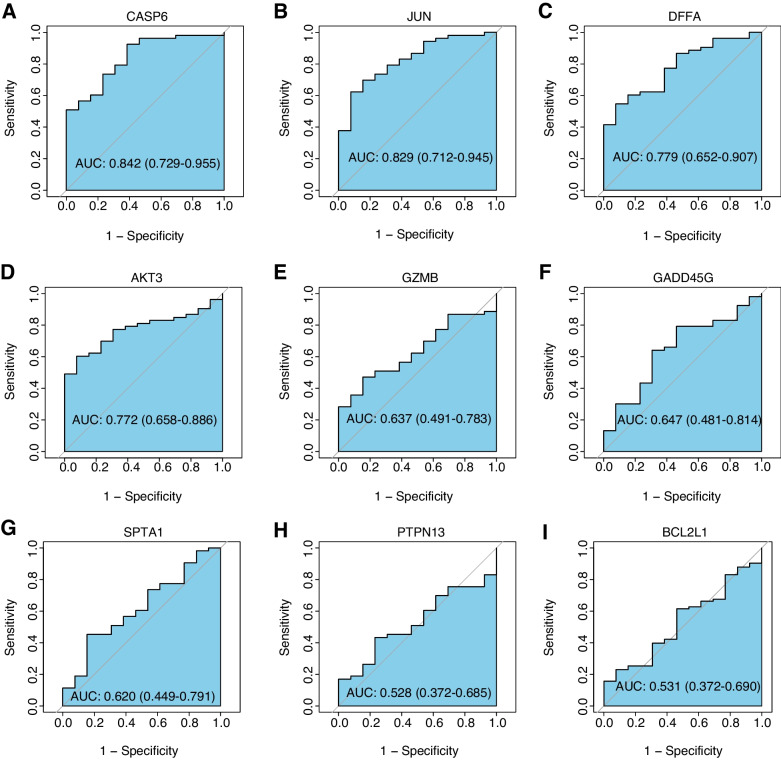
ROC analysis of the differentially-expressed apoptosis-related genes in GSE26155. **A** CASP6; **B** JUN; **C** DFFA; **D** AKT3; **E** GZMB; **F** GADD45G; **G** SPTA1; **H** PTPN13; **(I)** BCL2L1

136 apoptosis-related genes were retrieved from the KEGG’s apoptosis pathway (hsa04210). After screening them using | log2FC |> 0.5 and FDR < 0.05 as thresholds, we finally extracted 481 up-regulated and 221 down-regulated genes (Fig. 1A). There were 9 differentially expressed apoptosis-related genes in TAA and healthy samples (Fig. 1B), which were CASP6, GZMB, SPTA1, DFFA, BCL2L1, JUN, PTPN13, GADD45G, and AKT3 (Table 1). The heatmap and boxplot showed that CASP6, GZMB, DFFA, JUN, PTPN13, and AKT3 were significantly underexpressed in the TAAs group, while SPTA1, BCL2L1, and GADD45G were significantly overexpressed (Fig. 1 C-D). The correlation heatmap revealed that GADD45G was significantly negatively correlated with AKT3, CASP6, and DFFA. Positive correlations were found between SPTA1 and BCL2L1, AKT3 and PTPN13, and AKT3 and CASP6 (Fig. 1E). The ‘GOSemSim’ package of R was used to calculate the semantic similarity in GO terms among these nine genes. The higher the semantic similarity, the more important the role that the gene plays in the function. Our results suggested that GADD45G presented the highest functional similarities (Fig. 1F). The ROC curve results showed that the AUC values of the 9 differentially-expressed apoptosis-related genes were all greater than 0.65, with CASP6 and AKT3 having AUC values greater than 0.7 suggesting that the selected differentially-expressed apoptosis-related genes had high diagnostic potential (Fig. 2). The results of the above genes in the validation dataset GSE26155 verified the results of the training set. The AUC values of AKT3, CASP6, DFFA, GADD45G, and JUN in the validation dataset all exceeded 0.7, while the AUC values of BCL2L1 and PTPN13 ranged from 0.5–0.6 (Fig. 3).

### Functional enrichment analysis of DEGs in TAAs

**Fig. 4:**
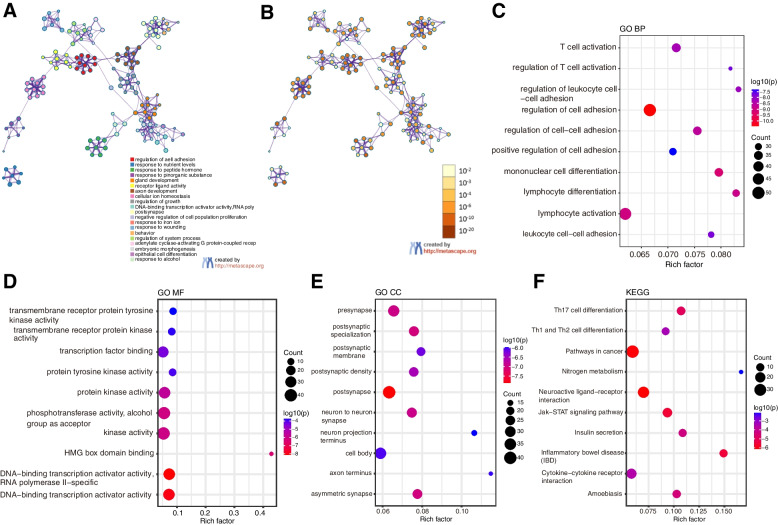
Functional enrichment analysis of DEGs. **A** Network diagram of enriched function, with different colors representing clusters of functional relevance; **B** Network diagram of enriched functions with color representing the *p*-values; **C** Top 20 GO biological process enriched terms; **D** Top 20 GO molecular function enriched terms; **E** Top 20 GO cellular compartment enriched terms; **F** Top 20 KEGG enriched pathways

GO and KEGG enrichment analyses of the 702 DEGs in TAAs were performed using the Metascape web tool to identify potentially significant biological functions related to apoptosis (Fig. 4) (Table 2). The results showed that the most significantly enriched GO terms included regulation of cell adhesion, lymphocyte activation, T cell activation, regulation of T cell activation (biological process), postsynapse, neuron-to-neuron synapse, neuron projection terminal, cellular component, DNA-binding transcription activator activity, transcription factor binding, transmembrane receptor protein kinase activity, and HMG box domain binding (molecular function). The results of KEGG enrichment analysis revealed that DEGs were mainly associated with inflammatory bowel disease (IBD), Th17 cell differentiation, Th1 and Th2 cell differentiation, Jak-STAT signaling pathway, cytokine-cytokine receptor interaction, and pathways in cancer.

**Table 2:** GO and KEGG enrichment analyses of the 702 DEGs between TAAs and Controls

Category	GO	Description	Count	LogP
GO Biological Processes	GO:0030155	regulation of cell adhesion	50	-10.4638
GO Biological Processes	GO:1903131	mononuclear cell differentiation	34	-9.24594
GO Biological Processes	GO:0046649	lymphocyte activation	48	-9.01933
GO Biological Processes	GO:0030098	lymphocyte differentiation	31	-8.90285
GO Biological Processes	GO:0022407	regulation of cell–cell adhesion	34	-8.65818
GO Biological Processes	GO:0042110	T cell activation	35	-8.2851
GO Biological Processes	GO:1903037	regulation of leukocyte cell–cell adhesion	28	-8.1533
GO Biological Processes	GO:0007159	leukocyte cell–cell adhesion	29	-7.80003
GO Biological Processes	GO:0050863	regulation of T cell activation	27	-7.71734
GO Biological Processes	GO:0045785	positive regulation of cell adhesion	31	-7.32084
GO Cellular Components	GO:0098794	postsynapse	40	-7.86597
GO Cellular Components	GO:0099572	postsynaptic specialization	27	-7.03801
GO Cellular Components	GO:0032279	asymmetric synapse	26	-6.99765
GO Cellular Components	GO:0098793	presynapse	33	-6.95219
GO Cellular Components	GO:0098984	neuron to neuron synapse	27	-6.91542
GO Cellular Components	GO:0014069	postsynaptic density	25	-6.55462
GO Cellular Components	GO:0045211	postsynaptic membrane	22	-6.18743
GO Cellular Components	GO:0044297	cell body	34	-6.06438
GO Cellular Components	GO:0043679	axon terminus	14	-6.00616
GO Cellular Components	GO:0044306	neuron projection terminus	15	-5.96515
GO Molecular Functions	GO:0001228	DNA-binding transcription activator activity, RNA polymerase II-specific	33	-8.10275
GO Molecular Functions	GO:0001216	DNA-binding transcription activator activity	33	-7.96265
GO Molecular Functions	GO:0008134	transcription factor binding	30	-4.33291
GO Molecular Functions	GO:0004713	protein tyrosine kinase activity	12	-3.93432
GO Molecular Functions	GO:0019199	transmembrane receptor protein kinase activity	12	-3.79084
GO Molecular Functions	GO:0004714	transmembrane receptor protein tyrosine kinase activity	11	-3.72145
GO Molecular Functions	GO:0071837	HMG box domain binding	6	-6.40631
GO Molecular Functions	GO:0016773	phosphotransferase activity, alcohol group as acceptor	38	-6.04666
GO Molecular Functions	GO:0016301	kinase activity	40	-5.97897
GO Molecular Functions	GO:0004672	protein kinase activity	33	-5.60797
KEGG Pathway	hsa05321	Inflammatory bowel disease	10	-5.49384
KEGG Pathway	hsa04659	Th17 cell differentiation	12	-4.92598
KEGG Pathway	hsa04658	Th1 and Th2 cell differentiation	9	-3.33179
KEGG Pathway	hsa04060	Cytokine-cytokine receptor interaction	19	-3.56502
KEGG Pathway	hsa04080	Neuroactive ligand-receptor interaction	26	-6.11963
KEGG Pathway	hsa05200	Pathways in cancer	34	-6.08129
KEGG Pathway	hsa04630	Jak-STAT signaling pathway	16	-5.57585
KEGG Pathway	hsa00910	Nitrogen metabolism	3	-2.10593
KEGG Pathway	hsa05146	Amoebiasis	11	-4.39642
KEGG Pathway	hsa04911	Insulin secretion	10	-4.25596

### Network reconstruction and functional analysis of apoptosis regulators and miRNA

**Fig. 5:**
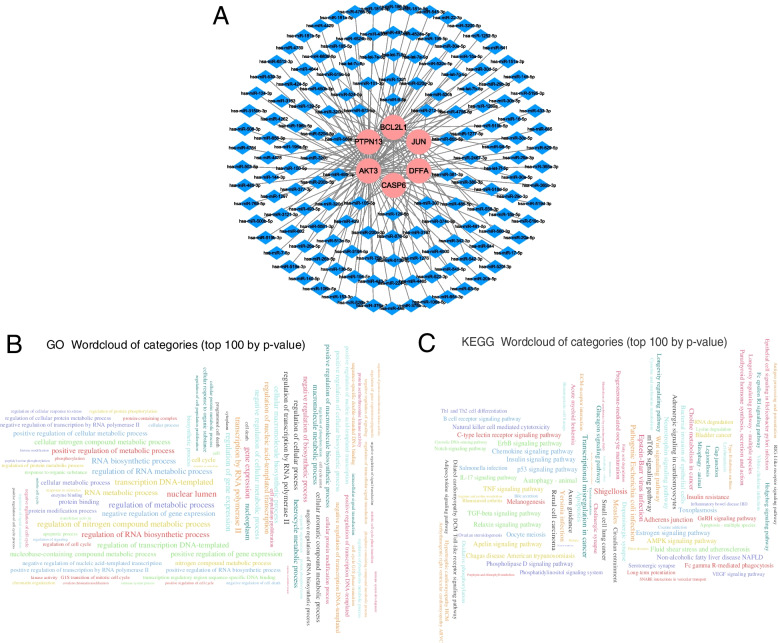
Interaction network analysis between apoptosis regulators and their targeted miRNAs. **A** mRNA-miRNA interaction network; **B** Wordcloud for significantly enriched GO terms; **C** Wordcloud for significantly enriched KEGG pathways

ENCORI and miRDB databases were used to identify mRNA-miRNA associations on 9 differentially-expressed apoptosis-related genes. Cross-linked miRNAs were selected to ensure the accuracy and stability of the results. 150 miRNAs and 6 mRNA-miRNAs regulatory networks mRNAs were obtained (Fig. 5A). Then, 150 miRNAs were used as input to miEAA for GO and KEGG enrichment analyses. The main enriched GO terms were positive regulation of the cellular biosynthetic process, positive regulation of the biological metabolic process, positive regulation of macroplated biosynthetic process, DNA-transcription temmolecule (process), protein binding, protein serinethreonine kinase activity, transferase activity (molecular function), nuclear lumen, nucleoplasm, and cytoplasm (cellular component) (Fig. 5B). The main enriched KEGG pathways were bacterial invasion pathways of epithelial cells, inflammatory bowel disease IBD, adherens junction, and IL-17 signaling pathway (Fig. 5C).

### Immuno-infiltration analysis

**Fig. 6:**
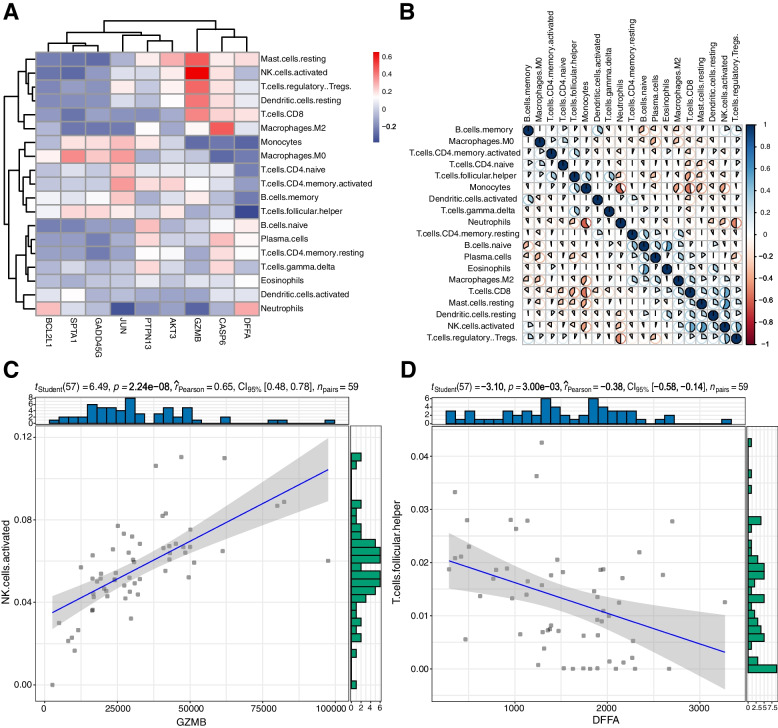
Immuno-infiltration analysis. **A** Correlation analysis between immune cell infiltration and differential apoptotic genes, where red and blue represented positive and negative correlation, respectively; **B** Correlation plot of immune cell infiltration, where red and blue represented positive and negative correlation, respectively. Color darkness was relative to the correlation strength; **C** the correlation among GZMB and activated NK cells; **D** the Correlation among DFFA and follicular helper T cells

The CIBERSORT algorithm was used for the quantification of the profiles of immune cells in the TME of TAA samples to study the relationship between apoptosis regulators and the immune microenvironment in TAA. As shown in Fig. 6B, heatmaps of 22 immune cells revealed that (1) Monocytes had a significant negative correlation with Neutrophils, Macrophages M2, T cells CD8, and activated NK cells; (2) T cell regulatory (Tregs) had a significant negative correlation with Neutrophils; (3) Eosinophils had a significant positive correlation with B cells naïve; (4) Activated NK cells had a significant positive correlation with T cells CD8 and Mast cells resting. Correlation analysis in Fig. 6A suggested that (1) GZMB presented a significant positive correlation with Mast cells resting, activated NK cells, T cell regulatory (Tregs), Dendritic cells resting, and T cells CD8, but had a negative correlation with Monocytes and Neutrophils; (2) CASP6 had a significant positive correlation with Macrophages M2, but had a significant negative correlation with Macrophages M0; (3) DFFA had a significant negative correlation with Monocytes, follicular helper T cells. Among them, GZMB presented the strongest positive correlation with activated NK cells (Fig. 6C), and DFFA had the strongest positive correlation with follicular helper T cells, respectively (Fig. 6D).

### Modification patterns mediated by apoptosis regulators

**Fig. 7:**
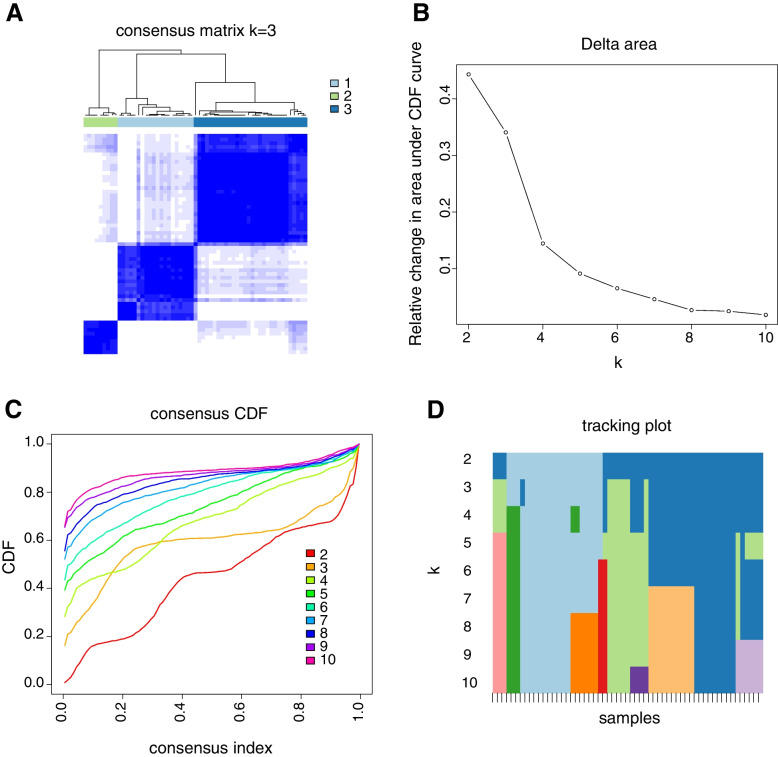
Unsupervised clustering analysis. **A** Consensus clustering plot at k = 3; **B** Cumulative distribution function (CDF) of consensus clustering; **C** Relative change in area under CDF curve from 2 to 10 of k; **D** Tracking plot

**Fig. 8:**
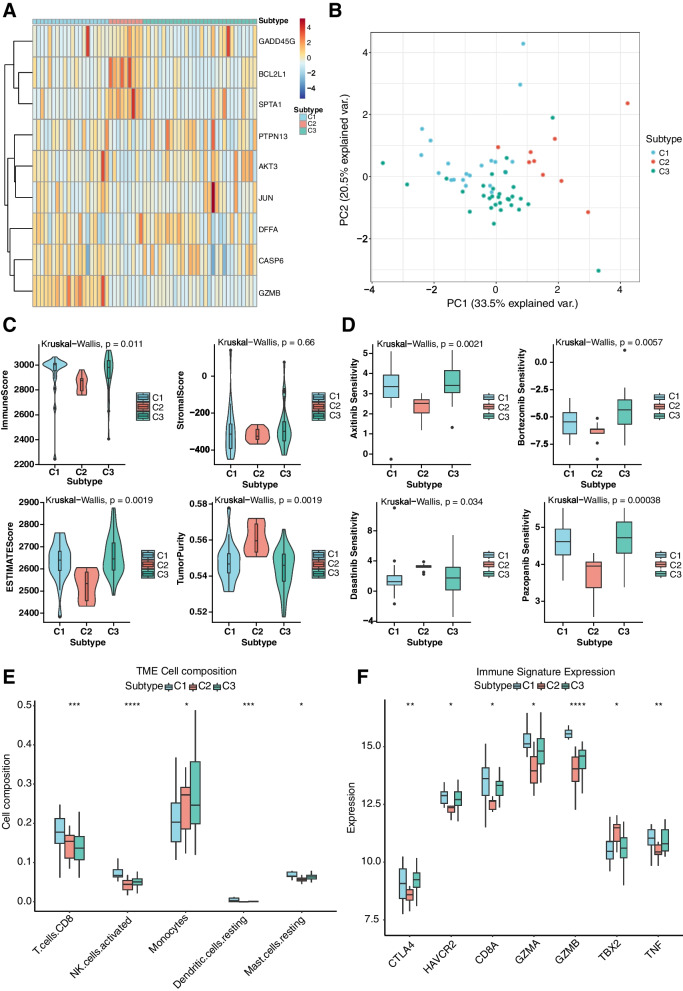
Mut omics of apoptosis modification patterns. **A** The unsupervised clustering of the 9 apoptosis regulators in the TAAs cohort. The apoptosis clusters were used for patients’ annotation. Red and blue colors represented high and low expression, respectively; **B** PCA of the transcriptomic profiles of the three apoptosis modification clusters, displaying evident diversity in the transcriptomic profiles between the different modification clusters; **C** Violin plots of ImmuneScore, StromalScore, ESTIMATEScore, and TumorPurity; **D** Drug sensitivity boxplots of Axitinib, Pazopanib, Bortezomib, and Dasatinib; **E** Boxplots of the proportions of immune cell infiltrates; **F** Boxplots of immune checkpoint and immunocompetent genes, * *p* < 0.05; ** *p* < 0.01; *** *p* < 0.001; **** *p* < 0.0001

The R package ConsensusClusterPlus was used to identify regulatory patterns of apoptosis genes through the relative expressions of nine differentially expressed apoptosis-related genes. Three different modification clusters were determined via unsupervised clustering (Fig. 7), including 20 patients with C1, 9 patients with C2 and 30 patients with C3. Nine differentially expressed apoptosis-related genes were significantly dysregulated in the revealed groups of patients (Fig. 8A-B), validating the existence of diversity apoptosis modification patterns in TAA. Immune infiltration, immune regulators and TIME were evaluated to study the differences in immune microenvironment characteristics among these distinct apoptosis modification patterns. ImmuneScore, StromalScore, ESTIMATEScore, and TumorPurity were obtained from ESTIMATE algorithms [21], and ImmuneScore, StromalScore, and ESTIMATEScore were the lowest in C2, while TumorPurity Score was the highest in C2 (Fig. 8C). According to the pRRophetic algorithm, we identified 4 chemotherapeutic drugs (i.e., Axitinib, Pazopanib, Bortezomib, and Dasatinib), that showed significant differences in drug sensitivity under the three regulatory patterns (Fig. 8D). Immune infiltration analysis (Fig. 8E) revealed that the fraction of T cells CD8 decreased from C1-C3. Besides, the activated NK cells and the resting Mast cells presented the lowest levels of immune infiltration in C2 and the highest levels of immune infiltration in C1. Monocytes had the highest levels of immune infiltration in C2 and the lowest levels of immune infiltration in C1. Dendritic cells resting had the highest levels of immune infiltration in C1. The boxplot of immune regulators (Fig. 8F) indicated that CTLA4, HAVCR2, CD8A, GZMA, GZMB, TBX2, and TNF were significantly dysregulated in three regulatory clusters. These analyses verified that apoptosis modification plays an essential regulatory role in shaping different immune microenvironments in TAA.

**Fig. 9:**
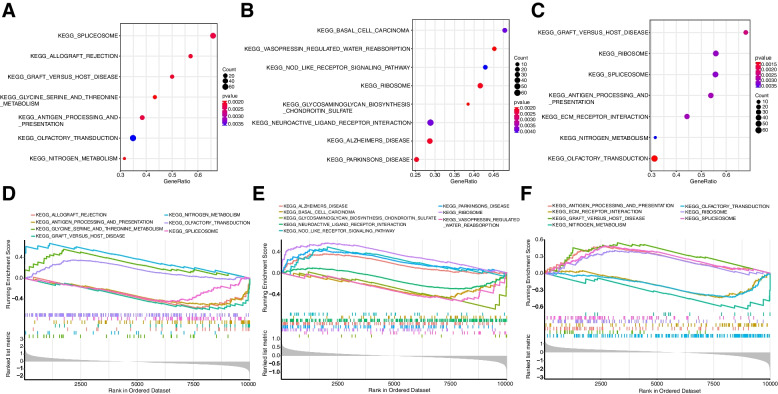
Biological features of apoptosis modification clusters. **A** Functional enrichment dotplot of C2 vs. C1; **B** Functional enrichment dotplot of C3 vs. C1; **C** Functional enrichment dotplot of C3 vs. C2; **D** Gseaplot of C2 vs. C1; **E** Gseaplot of C3 vs. C1; **F** Gseaplot of C3 vs. C2

GSEA analysis based on KEGG pathways was then performed to investigate the biological functions involved in apoptosis gene regulation clusters, and the results were as follows (Fig. 9) (Table 3): (1) C3 vs. C1 was mainly associated with Glycine, serine and threonine metabolism, Nitrogen Metabolism, Allograft rejection, and Graft-versus-host disease; (2) C2 vs. C1 was mainly associated with Glycosaminoglycan biosynthesis—chondroitin sulfate, Parkinson disease, Vasopressin-regulated water reabsorption, and Alzheimer disease; (3) C3 vs. C2 was mainly associated with Olfactory transduction, Graft-versus-host disease, Antigen processing and presentation, and ECM-receptor interaction.

**Table 3:** Gene set enrichment analysis among the 3 apoptosis modification clusters

C2 VS C1	Description	NES	Enrichment Score	*P*-value
KEGG Pathway	KEGG_GLYCINE_SERINE_AND_THREONINE_METABOLISM	1.838389	0.560845	0.001919
KEGG Pathway	KEGG_NITROGEN_METABOLISM	1.982373	0.673097	0.001957
KEGG Pathway	KEGG_ALLOGRAFT_REJECTION	-1.9107	-0.57536	0.002092
KEGG Pathway	KEGG_GRAFT_VERSUS_HOST_DISEASE	-2.01136	-0.60567	0.002092
KEGG Pathway	KEGG_ANTIGEN_PROCESSING_AND_PRESENTATION	-2.0796	-0.51944	0.002222
KEGG Pathway	KEGG_SPLICEOSOME	-1.87216	-0.45167	0.002227
KEGG Pathway	KEGG_OLFACTORY_TRANSDUCTION	1.634492	0.353626	0.003604
C3 VS C1	Description	NES	Enrichment Score	*P*-value
KEGG Pathway	KEGG_GLYCOSAMINOGLYCAN_BIOSYNTHESIS_CHONDROITIN_SULFATE	-1.83077	-0.68301	0.001934
KEGG Pathway	KEGG_VASOPRESSIN_REGULATED_WATER_REABSORPTION	-1.81291	-0.53842	0.001942
KEGG Pathway	KEGG_RIBOSOME	2.366854	0.563797	0.002033
KEGG Pathway	KEGG_PARKINSONS_DISEASE	1.874936	0.438462	0.00207
KEGG Pathway	KEGG_ALZHEIMERS_DISEASE	1.598138	0.358172	0.00211
KEGG Pathway	KEGG_NEUROACTIVE_LIGAND_RECEPTOR_INTERACTION	-1.41381	-0.30254	0.003817
KEGG Pathway	KEGG_BASAL_CELL_CARCINOMA	-1.73978	-0.4735	0.003854
KEGG Pathway	KEGG_NOD_LIKE_RECEPTOR_SIGNALING_PATHWAY	1.772937	0.486859	0.00409
C3 VS C2	Description	NES	EnrichmentScore	*P*-value
KEGG Pathway	KEGG_OLFACTORY_TRANSDUCTION	-1.88658	-0.42904	0.00129
KEGG Pathway	KEGG_GRAFT_VERSUS_HOST_DISEASE	1.85389	0.543605	0.002353
KEGG Pathway	KEGG_ANTIGEN_PROCESSING_AND_PRESENTATION	2.014427	0.485118	0.002976
KEGG Pathway	KEGG_ECM_RECEPTOR_INTERACTION	-1.66441	-0.43625	0.00299
KEGG Pathway	KEGG_RIBOSOME	1.733966	0.406238	0.003096
KEGG Pathway	KEGG_SPLICEOSOME	2.083989	0.488676	0.003175
KEGG Pathway	KEGG_NITROGEN_METABOLISM	-1.82152	-0.63843	0.003663

## Discussion

TAAs have a high prevalence worldwide and if undetected present high mortality, but their molecular mechanisms remain unclear [22]. Apoptosis is thought to be a carefully regulated energy-dependent process characterized by specific morphological and biochemical features in which caspase activation plays a central role. Growing evidence has suggested the crucial role of cell death in migration and invasion of disease [23]. Apoptosis refers to the process of programmed cell death. In this study, we revealed the expression of 9 apoptosis regulators which are significantly dysregulated between healthy and TAA, suggesting their involvement in TAA development. The co-expression analysis and GO semantic similarity analysis revealed the central role of GADD45G, suggesting that GADD45G might be a key molecule in the molecular regulatory mechanism of TAAs. GADD45G plays an essential regulatory role in DNA repair, cell cycle regulation, aging and genotoxic stress responses, and other cellular functions [24]. A large number of studies have shown that GADD45G is involved in the regulation of a variety of cell signaling pathways in tumor cells, and the significant reduction of their expression is closely related to tumor formation and progression [25–27]. The evaluation of the diagnostic potential of CASP6, AKT3, JUN, and DFFA measuring their AUC values revealed that they could discriminate TAAs and healthy samples with excellent specificity and sensitivity both in training and validation sets.

MiRNAs are endogenous non-coding RNA molecules that target the 3'UTR region of genes and can regulate gene expression by degrading or inhibiting the translation of target genes. Growing evidence have demonstrated that miRNAs can regulate apoptosis. C-JUN is a member of the JUN family and also a major component of the AP-1 transcriptional complex. Wang et al. proved that c-JUN triggers apoptosis in human vascular endothelial cells and c-JUN/AP-1 activation is an important mediator in endothelial cell apoptosis induced by oxidative stress, such as H2O2 [28]. MiR-29c exerts its effects on endometrial cell proliferation, apoptosis, and invasion by inhibiting the expression of c-JUN [29]. MiR-29b inhibits cell proliferation and invasion, and enhances cell apoptosis via targeting of AKT3 in prostate cancer [30]. Meanwhile, the up-regulation of miR-202-5p decreases the apoptosis of CML cells by lowering the level of CASP6 protein [31]. Functional enrichment analysis of the targeted mRNAs was then performed with results indicating that the targeted-by-miRNAs mRNAs were mainly enriched in the IL-17 signaling pathway, apoptosis, IBD, and TNF signaling pathway, chromatin organization, and cell death.

The tumor microenvironment (TME) mainly comprised various types of cells (immune cells, endothelial, fibroblastic, etc.) and extracellular components [32]. In the process of tumor apoptosis, the components of TME, such as immune cells, were often dysregulated. Apoptosis regulators were found to be related to immune infiltration, implying the essential role of apoptosis modification in TAA immune microenvironment regulation. Results showed that DFFA was negatively correlated with a follicular helper in T cells, and GZMB was positively correlated with activated NK cells, and CASP6 was positively correlated with M2 macrophages. Previous studies have revealed that GZMB was a member of the serine proteases family and expressed in granules of cytotoxic T-lymphocyte and NK cells [33]. The down-regulation of LINC02474 promoted the expression of GZMB, and the interference of GZMB could increase the metastatic abilities of colorectal cancer cells while reducing apoptosis [34]. Interrogans-induced apoptosis in macrophages is mediated by CASP3 and CASP6 activation through a Fas-associated death domain (FADD)-CASP8-dependent pathway [35]. As a substrate of caspase-3 that can trigger DNA fragmentation during apoptosis, DFFA’s expression is inhibited in tumor cells to varying degrees, including gastrointestinal cancer, bladder cancer, and so on [36, 37]. These findings could point out the direction of the apoptosis immune regulation mechanism in TAA.

In addition, we identified the regulatory patterns/clusters of the apoptosis genes (i.e., C1, C2, C3) by consensus clustering based on the expression of nine differential apoptosis genes in TAAs. The regulatory clusters based on the apoptosis genes showed significantly different RNA epigenetics, immune status, chemosensitivity, biological processes and outcomes. Patients in the C2 group had the lowest ImmuneScore, StromalScore, and ESTIMATEScore, but the highest TumorPurity. Four drugs, axitinib, pazopanib, bortezomib, and dasatinib, displayed significant differences in drug sensitivity in the 3 different regulatory clusters. Immune infiltration and immune regulators’ expression significantly differed among the three regulatory clusters C1, C2 and C3. The results of GSEA functional enrichment suggested that the regulatory clusters were enriched in different KEGG pathways. Studying the biological characteristics of each subtype, confirmed the reliability of our revealed TAA sub-phenotypes based on apoptosis genes expression profiles. This classification strategy for the apoptosis subtype can help us understand the underlying mechanism of apoptosis regulation so that precise therapeutic methods can be applied. TAA can thus be subtyped from the molecular level or immune level and not only from the phenotype of the patients. To our knowledge, this is the first study on transcriptome-wide mapping of apoptosis genes, which focuses on investigating the landscape and function of reversible RNA modifications in TAAs.

The typical histological manifestations in TAA are Elastic fiber fragmentation and disarray, often with a concomitant depletion of vascular smooth muscle cells (VSMCs). Both inflammation and TGFβ dysregulation are considered triggers of VSMC apoptosis, as apoptotic cells in the aortic wall collocate with inflammatory cells and the overexpression of TGFβ. The apoptosis of VSMCs further leads to an increase in angiotensin II signaling, shear stress, reactive oxygen species formation, and imbalances of pro- and antiapoptotic factors of the BCL2 protein family [38]. BCL2L1 is an anti-apoptotic BCL2-family member. Moreover, Liu et al. revealed that TGF-β could skew macrophage polarization towards the M2-like phenotype, improve the phagocytic ability via the AKT/FoxO1 pathway, and reduce inflammatory reactions in sepsis [39]. Therefore, the dysregulation of the TGFβ pathway may lead to the occurrence of TAA.

The significance of this study is not only to fill the gap in the study of TAAs apoptosis-related genes and to continue to enrich and improve the study of TAAs diagnostic markers but also to provide some theoretical guidance in the field of TAAs clinical treatment and advance the study of TAAs immunotherapy providing the required theoretical background. Although some results were obtained in terms of apoptosis-related genes in TAAs cells, there are still some limitations. First, the data in the training set came from the peripheral blood, while the data in validation set came from the aortic wall. However, Peripheral blood cells (PBCs) gene expression profiles have been used to identify signatures for autoimmune diseases and cardiovascular diseases, such as systemic lupus, rheumatoid arthritis, multiple sclerosis, atherosclerosis, and coronary artery disease [40–45]. In addition, the results obtained from our validation are also satisfactory. Nevertheless, further experiments are required to verify the diagnostic potential of the screened molecules. Second, the apoptosis genes that were screened proved to be of clinical value, but we did not establish a scoring system or did not explore their use for translational application and research. Therefore, we suggest as an interesting future work to collect additional TAAs data, carry out relevant experimental verification, and construct a more accurate clinical scoring system to provide the necessary reference for the precise treatment of TAAs.

## Conclusions

In summary, the present manuscript identified through bioinformatics analysis 9 potential apoptosis-related genes dysregulated between TAAs and samples from healthy individuals. These novel genes may affect the development of TAA by regulating apoptosis. Our study also revealed the underlying regulatory mechanisms of apoptosis modification in TAAs. The comprehensive analysis of apoptosis genes and apoptosis modification clusters is a great contribution to understanding the potential mechanism of the apoptosis regulation network in TAA, inspiring more effective therapeutic methods.

## Data Availability

Publicly available datasets were analyzed in this study. The data can be found in GEO, https://www.ncbi.nlm.nih.gov/geo/ (GSE9106).
